# Retinal Nerve Fibre Layer Thinning in Patients with Clinically Isolated Optic Neuritis and Early Treatment with Interferon-Beta

**DOI:** 10.1371/journal.pone.0051645

**Published:** 2012-12-13

**Authors:** Kurt-Wolfram Sühs, Katharina Hein, Jens R. Pehlke, Barbara Käsmann-Kellner, Ricarda Diem

**Affiliations:** 1 Department of Neurology, Saarland University, Homburg, Germany; 2 Department of Psychiatry, Social Psychiatry and Psychotherapy, Hannover Medical School, Hannover, Germany; 3 Department of Neurology, Georg-August University, Göttingen, Germany; 4 Department of Addiction Disorders, LWL Clinic Münster, Münster, Germany; 5 Department of Ophthalmology, Saarland University, Homburg, Germany; 6 Department of Neuro-oncology, University Clinic Heidelberg, Heidelberg, Germany; Friedrich-Alexander University Erlangen, Germany

## Abstract

**Background:**

Optic neuritis is associated with neurodegeneration leading to chronic impairment of visual functions.

**Objective:**

This study investigated whether early treatment with interferon beta (IFN-β) slows retinal nerve fibre layer (RNFL) thinning in clinically isolated optic neuritis.

**Methods:**

Twenty patients with optic neuritis and visual acuity decreased to ≤0.5 (decimal system) were included into this prospective, open-label, parallel group 4-month observation. After methylprednisolone pulse therapy, 10 patients received IFN-β from week 2 onwards. This group was compared to 10 patients free of any disease modifying treatment (DMT). The parameter of interest was change in RNFL thickness assessed at baseline and at weeks 4, 8, and 16. Changes in visual acuity, visual field, and visual evoked potentials (VEPs) served as additional outcome parameters.

**Results:**

RNFL thinning did not differ between the groups with a mean reduction of 9.80±2.80 µm in IFN-β-treated patients (±SD) vs. 12.44±5.79 µm in patients who did not receive DMT (baseline non-affected eye minus affected eye at week 16; p = 0.67, t-test, 95% confidence interval: −15.77 to 10.48). Parameters of visual function did not show any differences between the groups either.

**Conclusions:**

In isolated optic neuritis, early IFN-β treatment did not influence RNFL thinning nor had it any effect on recovery of visual functions.

## Introduction

Multiple sclerosis (MS) is a major cause of neurological disability in young adults and often manifests with optic neuritis as its earliest clinical presentation. In both MS and optic neuritis, neurodegenerative changes lead to persistent functional deficits. [1], [2] Disease modifying therapy (DMT) with interferon-beta (IFN-β) belongs to the standard therapies of MS and has already been found to be beneficial in an early disease stage termed clinically isolated syndrome (CIS). [3]–[6] However, irrespective of the documented effects on immune responses, it still is a subject of discussion whether and to what extent DMT counteracts inflammation-associated neurodegeneration. [2] Although treatment with IFN-β has been shown to delay conversion of CIS into clinically definite MS, neurological disability was not reduced five years later [4] suggesting that neurodegeneration might not be influenced by early start of DMT. Direct monitoring of neurodegeneration in patients with CIS is challenging, particularly due to the heterogeneity of this patient group with different anatomical lesion localisation, variable numbers of initial lesions, and different dynamics of neurodegeneration.

Amongst the spectrum of CIS, optic neuritis represents a homogenous disease condition with a predictable extent of retinal nerve fibre layer (RNFL) degeneration. By using optical coherence tomography (OCT), atrophy of the RNFL which contains the non-myelinated part of optic nerve axons can directly be assessed. [7] Moreover, changes in RNFL thickness during MS allow conclusions to be drawn about neurodegenerative changes on a global level due to their association with brain atrophy. [8]–[13] In previous work, it has been shown that RNFL thinning can be observed during the first six months following optic neuritis and that more than 90% of this fibre loss has occurred by the end of three months. [14] Additionally, in a recent study testing erythropoietin in optic neuritis, we had shown that an observational period of 16 weeks is appropriate to cover RNFL changes following optic neuritis and to detect potential effects of neuroprotective treatment. [15].

In the present study, we have chosen optic neuritis as a homogenous presentation of CIS in order to investigate neuroprotective effects of the most frequently prescribed DMT. Following optic neuritis, the time-course of RNFL thinning after early start of IFN-β therapy was compared to that of a group of untreated patients. In order to assess potential effects of early DMT on recovery of visual function, we additionally investigated changes in visual acuity, visual field, and latencies and amplitudes of visual evoked potentials (VEPs).

## Methods

### Patients and Therapy

Patients between 18–50 years of age having presented at Saarland University Clinic (Germany) with optic neuritis as CIS were eligible to be included into this parallel group observation. Onset of symptoms had to be within the previous 10 days and visual acuity as assessed by an ophthalmologist had to be decreased to 0.5 (decimal system) or less. Please note that a decimal value of 0.5 corresponds to a logarithmic (log) visual acuity of −0.3, a minimum angle of resolution (MAR) of 2.0, a logMAR value of 0.3, or a Snellen equivalent of 6/12 (with metres used instead of feet). Treatment with subcutaneously applied IFN-β1b (250 µg every other day; Betaferon®) or IFN-β1a (44 µg three times a week, Rebif®) was offered to all patients in agreement with the licensed use of the drug if oligoclonal bands were identified in the cerebro-spinal fluid (CSF) following routinely performed lumbar puncture, and/or at least one clinically silent demyelinating brain lesion was detected by magnetic resonance imaging (MRI). Therefore, patients were identified to be at increased risk for the development of MS. [16]–[19] Treatment was started within one week after methylprednisolone pulse therapy (1000 mg i.v. per day for three days). To reduce the risk of side effects, the dosage of IFN-β was gradually increased reaching full dosage within the first four weeks. Routine treatment with any other of the available IFN-β-containing products or with glatiramer acetate was offered as an alternative to study participation. Patients who did not wish to receive DMT were offered to be included into the non-treatment arm of this observational study. Prior to inclusion patients gave informed consent.

Exclusion criteria consisted of any ocular disease (affected or non-affected eye), visual acuity of <1.0 in the non-affected eye, significant hyperopia, myopia, or astigmatism, pregnancy, lactation period, or any disease interfering with the use of corticosteroids or IFN-β. Immunosuppressive or immunomodulatory pre-treatments were not allowed at any time as well as treatment with corticosteroids within 30 days prior to inclusion. The study was approved by the Ethics Committee of the University of the Saarland.

### Procedures and Outcome Measures

OCT was performed with a time domain OCT (Stratus OCT Model 3000; Carl Zeiss Meditec, Jena, Germany) using the fast RNFL thickness protocol. OCT software employed an algorithm to calculate the average thickness of the RNFL, and to compare these measurements to a database of age-matched controls. Mean overall, quadrant, and sector RNFL measurements were obtained. Intra-class correlation coefficients (determined by using type 3,1 of the intracc.sas macro, http://euclid.psych.yorku.ca/ftp/sas/macros/intracc.sas) for repeated OCT measurements of a control population of 15 healthy, age- and gender-matched subjects were in the range of 0.81 indicating good reproducibility of the measurements. Refractive errors were determined by retinoscopy and automated refractometry. After subjective refraction, the best correction was used to determine the distant visual acuity according to DIN58220 (Landolt rings, forced choice, 3/5 criterion). Near visual acuity was determined using the OCULUS text chart. Visual acuity was described according to the decimal system. The central 60° visual field (radius 30°) was measured by static automated perimetry using the program G1X of the OCTOPUS perimeter (INTERZEAG Switzerland). Perimetric results were analyzed with respect to age-related data and changes were calculated by the OCTOPUS software.

Recordings and analyses of VEPs were performed as described previously. [20] In cases of severe optic neuritis manifested by an inability to respond to VEP stimulation or visual testing, values of 170 ms and 0 µm were given for latency and amplitude [21], and a value of 0 was given for visual acuity.

At each of the visits, patients underwent neurological examinations to assess disability according to the widely and routinely used Expanded Disability Status Scale (EDSS). Additionally, MRI was performed at baseline. For MRI, a 1.5T machine (Siemens Magnetom Sonata, Siemens, Erlangen, Germany) was used. The following images were obtained: 3 mm contiguous slices from transversal PD/T2-weighted turbo spin echo, from sagittal T2-weighted turbo spin echo, and from a sagittal T1-weighted 3D-sequence with application of gadolinium-diethylene triamine pentaacetic acid in a body weight-adapted dosage.

OCT, recordings of VEPs, and assessments of visual acuity and visual field parameters were performed according to established time-points for follow-up which have been shown to be appropriate for neuroprotection trials in optic neuritis. [15] These time-points include weeks 4, 8, and 16. RNFL thickness of the affected eye at week 16 was subtracted from the baseline value of the affected eye or, in an alternative analysis, from the baseline value of the non-affected eye in order to estimate the impact of initial RNFL swelling. Respectively, the amount of RNFL thinning over 16 weeks was compared between the groups. Additionally, we compared recovery of visual acuity and visual field, and changes in latencies and amplitudes of VEPs over the 16 week observation period in the affected eye. Comparison of absolute values at week 16 of the affected eyes was also performed in addition to comparison of differences between the non-affected and affected eye in each patient.

### Statistical Analysis

A total of 20 patients were assigned to this observational study (10/10 IFN-β/no DMT) and underwent intention-to-treat (ITT) analysis. The ITT analysis included all patients who had baseline assessment and at least one follow-up. Six patients had incomplete datasets and missing values were replaced by the LOCF (“last observation carried forward”) method. Analyses were performed using student’s t-test with assessment of 95% confidence intervals (CI). Furthermore, we performed an analysis of covariance (ANCOVA) in order to test whether baseline differences may have any effects on the results of the primary outcome parameter.

## Results

### Baseline Characteristics and Clinical Disease Course

Demographics, time to inclusion after symptom onset and baseline characteristics of the two groups (IFN-β/no DMT) consisting of 10 patients each are given in Tables 1 and 2. At baseline, mean VEP latencies were longer in patients which were assigned to the IFN-β treatment group (161.1±4.072) compared to untreated patients (144.1±6.414; ±SD; p = 0.033; 95% CI 1.592 to 32.57) indicating potentially more severe optic neuritis in this group. As another indication for differences in general disease severity, five patients out of the IFN-β group had more than 9 T2-weighted MRI lesions, whereas in the non-DMT group, it was only one. None of the other outcome parameters showed significant baseline differences. Mean EDSS at baseline was 3.1±0.75 (±SD) in patients who received later IFN-β treatment and 3.0±0.58 in patients of the non-DMT group. During the observation period of 16 weeks, one patient in the IFN-β group and one patient without DMT experienced a second clinical relapse and was diagnosed with MS. As a consequence, treatment with IFN-β was initiated in the latter but since first dose was not applied before week 14, for final analysis this patient was still considered to be “untreated”. The frequency and spectrum of undesired drug effects was within the established tolerability profiles of methylprednisolone or IFN-β.

**Table 1:** Demographic data and baseline characteristics.

	IFN-β (n = 10)	no DMT (n = 10)	p value (t-test)
**Age (years)**	41.1 (±5.43), 43.5	35.5 (±7.38), 33.5	–
**Women**	7 (70%)	8 (80%)	–
**Days after onset of symptoms**	5.3 (±2.1), 6.0	5.7 (±2.4), 7.0	–
**Visual acuity (decimal system)**	0.19 (±0.07), 0.105	0.24 (±0.07), 0.2	0.59
**RNFL thickness (µm),** **affected eye**	102.40 (±5.11), 98.5	113.10 (±10.76), 109.0	0.38
**VEP latency (ms)**£	161.1 (±4.07), 170.0	144.1 (±6.41), 144.7	0.033*
**VEP amplitude (µV)**£	2.23 (±1.17), 0.0	3.31 (±0.76), 2.7	0.49
**Volume of scotoma (dBdeg^2^)**£	31970 (±6970), 24622	18620 (±7210), 11220	0.21

Data are mean (± SD), number (%), or median. Parameters at baseline were compared by using t-test. Respective p values are indicated. £Data available for 10 patients in the IFN-β group and 8 patients in the group not having received DMT. RNFL, retinal nerve fibre layer; IFN-β, interferon-β; DMT, disease modifying treatment; dB, decibel.

**Table 2:** T2-weighted brain MRI lesions at baseline.

(n = 10)	1–4 T2w	5–9 T2w	10–19 T2w	≥20 T2w
**IFN-β**	3	2	3	2
**no DMT**	3	6	1	–

Data represent absolute patient numbers. Presence of at least one clinically silent T2w brain lesion was one of the inclusion criteria of the study. Data available for 10 patients in the IFN-β group and 10 patients in the group not having received DMT. T2w, T2-weighted; IFN-β, interferon-β; DMT, disease modifying treatment.

### Changes in Retinal Nerve Fibre Layer Thickness

**Figure 1:**
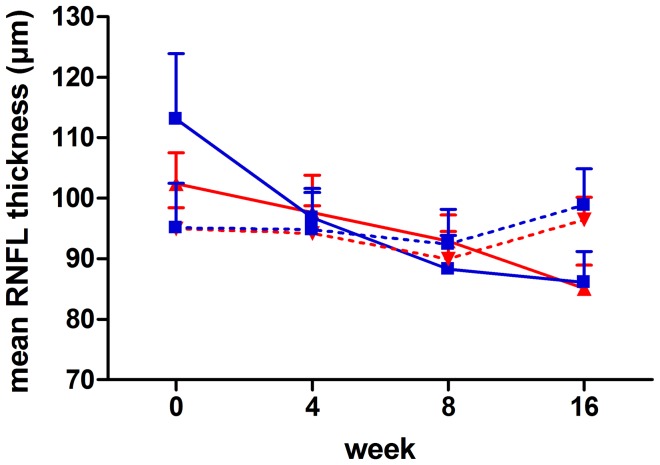
Intention-to-treat analysis of the primary parameter of interest. Mean RNFL thickness at the different time-points given in relation to RNFL thickness of the contralateral healthy eye. Red line = IFN-β, affected eye; blue line = no DMT, affected eye; interrupted red line =  IFN-β, healthy eye; interrupted blue line = no DMT, healthy eye; Bars = SEM.

After LOCF substitution of values which was performed in a total of 6 patients (2 IFN-β/4 non-DMT), the ITT analysis included 20 patients of whom 10 had received early IFN-β therapy and 10 belonged to the non-DMT group. All patients whose data were substituted by LOCF had complete data except from the final visit. From baseline to week 16, RNFL thickness of the affected eyes decreased by a mean (±SD) of 17.3±2.83 µm in IFN-β-treated patients (median 16.5 µm) and by 29.22±12.37 µm (median 20.0 µm) in patients not having received DMT (p = 0.36, t-test; 95% confidence interval −38.59 to 14.75; Table 3). Figure 1 shows the time-course of RNFL thinning in relation to RNFL thickness values of the contra-lateral healthy eyes indicating that RNFL oedema was present at baseline with resolution being completed between weeks 6–8. To control for effects of IFN-β on resolution of oedema, we additionally applied another type of analysis comparing RNFL differences of baseline non-affected eyes to affected eyes at week 16 between the groups (RNFL non-affected eye at baseline minus RNFL affected eye at week 16; Table 3). This type of analysis was done in order to use baseline RNFL thickness of the non-affected eye as an estimate of what baseline RNFL thickness in the affected eye would have been. The results of this comparison also did not reveal any statistically significant differences between the groups. In IFN-β-treated patients, subtracting RNFL thickness of the affected eyes at week 16 from baseline RNFL thickness of the non-affected eyes revealed a mean of 9.80±2.80 µm compared to 12.44±5.78 µm in patients not having received DMT (p = 0.67, 95% confidence interval −15.77 to 10.48). Absolute RNFL thickness at week 16 did not significantly differ between the groups (IFN-β: 85.1±3.87 µm vs. non-DMT: 86.1±5.14 µm; p = 0.8781; 95% confidence interval −14.51 to 12.51) nor did the results of the intra-individual comparison at week 16 (affected vs. non-affected eye; table 3). RNFL thickness of the non-affected eye did not change over the observation period.

**Table 3:** Intention-to-treat comparison of RNFL thicknesses and rates of change.

	IFN-β (n = 10)	no DMT (n = 10)	t-test (95% CI)
**Baseline RNFL thickness (absolute values; µm)**	102.4 (±5.11), 98.5	113.1 (±10.76), 109.0	0.38 (−35.72–14.32)
**Difference between non-affected and affected eye at baseline (µm)**	−7.5 (±3.91), −9.5	−15.78 (±11.72), −13.0	0.49 (−16.67–33.22)
**RNFL thickness at week 16 (absolute values; µm)**	85.10 (±3.87), 86.0	86.10 (±5.14), 82.5	0.88 (−14.51–12.51)
**Difference between non-affected and affected eye at week 16 (µm)**	11.3 (±2.84), 10.0	11.6 (±2.91), 11.0	0.94 (−8.856–8.256)
**RNFL thinning over 16 weeks (µm) (baseline affected eye minus affected eye at week 16)**	17.30 (±2.83), 16.5	29.22 (±12.37), 20.0	0.36 (−38.59–14.75)
**RNFL thinning over 16 weeks (µm) (baseline non-affected eye minus affected eye at week 16)**	9.80 (±2.80), 8.0	12.44 (±5.78), 6.0	0.67 (−15.77–10.48)

Data are mean (±SD) or median. Ten patients per group underwent intention-to-treat analysis. RNFL, retinal nerve fibre layer; IFN-β, interferon-β; DMT, disease modifying treatment; CI, confidence interval.

### Recovery of Visual Acuity and Visual Field Perception

**Figure 2:**
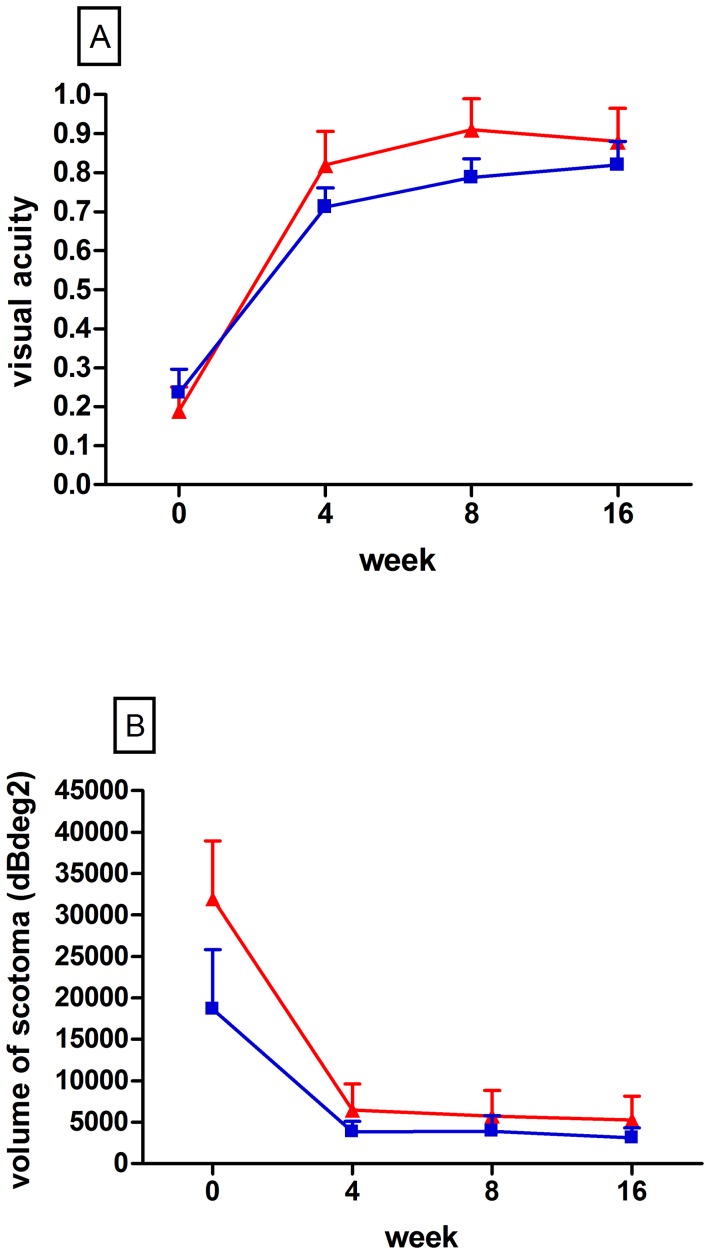
Intention-to-treat analyses of additional outcome parameters. Visual acuity (**A**) and visual field deficits (**B**) under treatment with IFN-β (red line) or non-DMT (blue line). Visual acuity is expressed according to the decimal system. Bars = SEM.

Mean visual acuity in IFN-β-treated patients recovered from a baseline value of 0.19±0.07 (±SD; decimal system; median, 0.105) to 0.88±0.08 at week 16 (median, 1.0) corresponding to a change of 0.69±0.33 over the observation period (mean±SD; n = 10; ITT analysis). A similar recovery of vision was observed in the group of patients which did not receive DMT. In these patients, visual acuity improved from a baseline value of 0.24±0.07 (median, 0.2) to 0.82±0.06 (median, 0.85) at week 16 with a respective change of 0.57±0.19 (mean±SD; n = 10; ITT analysis; p = 0.30 when compared to IFN-β-treated patients; t-test; 95% confidence interval −0.1256 to 0.3816; Fig. 2A). Absolute values at week 16 were also not significantly different between the groups (p = 0.55, t-test). Figure 2A shows the time-course of functional recovery in both patient groups with more than 90% of improvement having occurred by week 4.

In parallel to the recovery of visual acuity, visual field deficits improved in both groups of patients with the highest amount of recovery also taking place within the first four weeks (Fig. 2B). In IFN-β-treated patients (n = 10), the volume of scotoma recovered from 31970±6970 dBdeg^2^ at baseline to 5272±2892 dBdeg^2^ at week 16 (mean±SD; median baseline, 24622 dBdeg^2^; median week 16, 875 dBdeg^2^) with a change of −26110±5508 dBdeg^2^ over the time-course of the study. In the group which did not receive DMT, visual field data was not assessed in two of the patients leaving eight patients for analysis of visual field deficits in this group. The volume of scotoma in these patients changed from 18620±7210 dBdeg^2^ at baseline to 3084±1252 dBdeg^2^ at week 16 (median baseline, 11220 dBdeg^2^; median week 16, 1176dBdeg^2^) resulting in a change of −15360±7558 dBdeg^2^ over 16 weeks (p = 0.26 when compared to the IFN-β-treated group, t-test; 95% confidence interval −30310 to 8805). Absolute values at week 16 did not significantly differ either (p = 0.53, t-test).

### Changes in Latency and Amplitude of Visual Evoked Potentials

**Figure 3:**
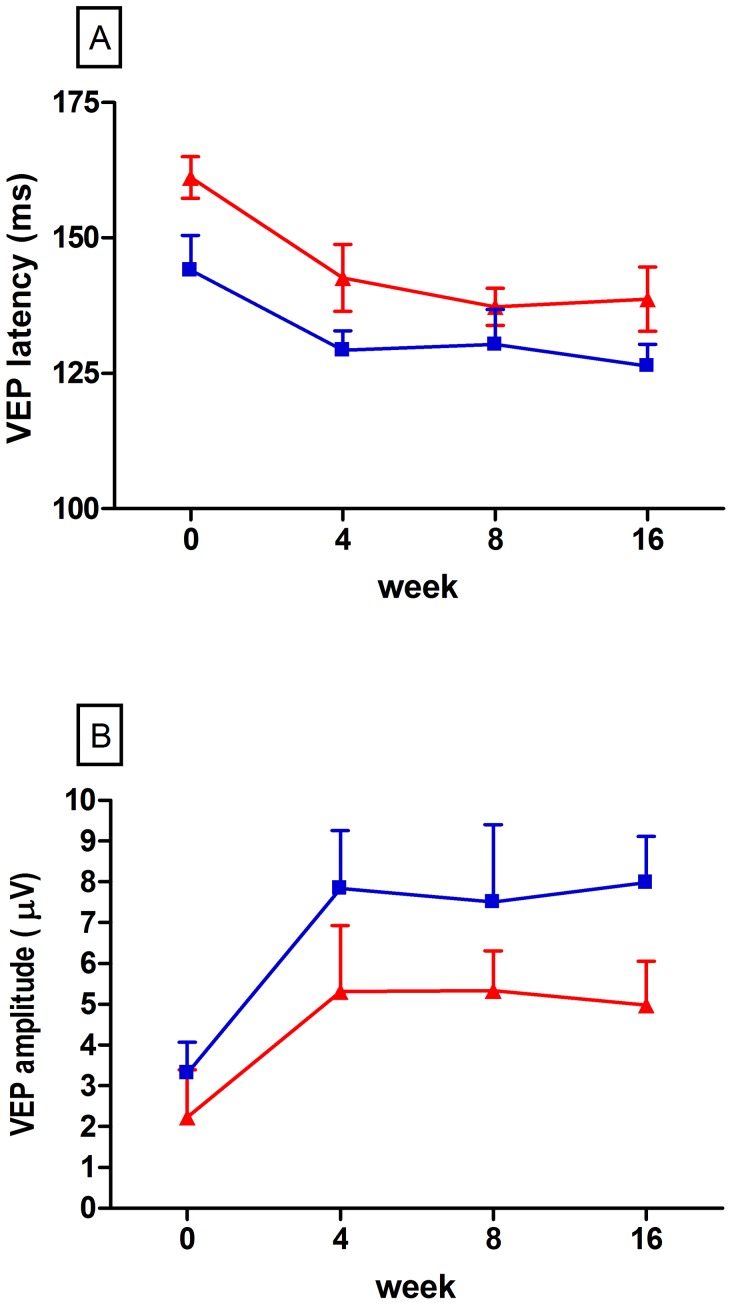
Intention-to-treat analyses of VEP amplitudes and latencies. VEP latency (**A**) and amplitude (**B**) under treatment with IFN-β (red line) or non-DMT (blue line). Bars = SEM.

At baseline, VEP latencies were prolonged in both groups of patients (see table 1). Although patients with later IFN-β treatment had longer VEP latencies at baseline, rates of change over the study duration were similar in both groups (Fig. 3A). Patients with IFN-β treatment showed a mean latency of 138.7±6.274 ms at week 16 (n = 10; median, 133.7 ms) with a mean reduction of 22.48±19.18 ms over 16 weeks whereas in the group not receiving DMT, mean latency at week 16 was 126.3±4.041 ms (n = 8; median, 120.6 ms) resulting in a reduction of 14.51±10.04 ms (mean±SD; p = 0.35, t-test; 95% confidence interval −9.781 to 25.71). Comparison of absolute latencies at week 16 did not reveal any significant differences either (p = 0.14, t-test). Co-variable testing in order to assess the influence baseline VEP latency exerts on RNFL thickness at week 16 exerted a p value of 0.209 indicating a weak or even non-existent effect of this co-variable.

In parallel to the time-course of latency reduction, VEP amplitudes increased (Fig. 3B). Again, rates of change over the 16 week observational period were similar between the groups (mean change±SD IFNβ, 2.76±0.99 µV vs. non-DMT, 4.17±1.07 µV; n = 10 for IFNβ n = 8 for non-DMT; p = 0.36, test; 95% confidence interval −4.570 to 1.747). Absolute values at week 16 were 4.99±1.08 µV (mean±SD; IFN-β; median, 5.70 µV) and 7.988±1.14 µV (non-DMT; mean, 7.35 µV), respectively, and did not significantly differ (p = 0.08, t-test). In addition, differences in VEP latency and VEP amplitudes between the non-affected and the affected eye were comparable among the groups (Table 4).

**Table 4:** Comparison of VEP amplitudes and latencies and rates of change.

	IFN-β (n = 10)	no DMT (n = 8)	t-test (95% CI)
**Difference in VEP amplitude between non-affected and affected eye at baseline**	6.17 (±1.74), 5.0	4.83 (±1.46), 3.1	0.59 (−1.58–9.04)
**Difference in VEP amplitude between non-affected and affected eye at week 16**	4.48 (±1.83), 2.8	0.75 (±1.61), 1.6	0.16 (−16.67–33.22)
**Difference in VEP latency between non-affected and affected eye at baseline**	−52.01 (±5.85), −47.45	−39.84 (±6.51), −42.30	0.18 (−30.74–6.39)
**Difference in VEP latency between non-affected and affected eye at week 16**	−29.47 (±6.07), −24.90	−21.05 (±4.05), −19.65	0.29 (−24.78–7.94)

Data are mean (±SD) or median. VEP, visual evoked potentials; RNFL, retinal nerve fibre layer; IFN-β, interferon-β; DMT, disease modifying treatment; CI, confidence interval.

## Discussion

In this observational study, we have assessed the effects of early IFN-β therapy on RNFL degeneration and recovery of visual function in patients following isolated severe optic neuritis with increased risk of developing MS. We found that starting treatment with IFN-β within one week after methylprednisolone pulse therapy did not influence the amount of RNFL atrophy when compared to untreated patients nor did it improve recovery of functional parameters of vision.

This study was performed in order to address the question whether or not early DMT exerts neuroprotective effects in optic neuritis presenting as CIS. Under these disease conditions, neuroprotection induced by DMT appears to be conceivable since neurodegenerative processes in optic neuritis and MS have been shown to occur early [14], [22] and to depend on inflammatory changes [23]. Observations of increased nerve growth factor concentrations in glial and brain endothelial cell cultures after exposure to IFN-β [24], [25] together with findings of increased neuronal survival *in vitro* [26] further support this assumption. In clinical trials, effects of IFN-β on general brain atrophy as indirect evidence for neuroprotection have been described [27], however, these effects were inconsistently seen for different formulations of IFN-β and reflect the sum of various tissue changes such as oedema resolution or demyelination. [28] Assessment of RNFL thinning in contrast allows direct investigation of neuroaxonal retinal damage [7] and has recently been demonstrated to be a useful primary outcome parameter for neuroprotection trials in optic neuritis. [15] By comparing with RNFL thickness values of the contralateral healthy eyes, the time to oedema resolution can be determined and distinguished from atrophy of the RNFL caused by nerve fibre loss itself. In agreement with our previous observations [15], RNFL oedema in both of the groups studied here has been resolved within the first two months after disease onset with further RNFL thinning to levels more than 10 µm below those of the corresponding healthy eyes. Absolute RNFL thickness at week 16 in both of our groups was at a similar range to previously published 6-month values in patients after optic neuritis according to estimates derived from an exponential model which suggests 90% of RNFL thinning to be reached 2.38 months after disease onset and 99% of total RNFL loss to occur by a mean time of 4.75 months. [14] However, as particularly revealed by longitudinal follow-up on unaffected eyes in both of our patient groups, some extent of intra-individual (intra-subject) variation occurs. This variation might be diminished by applying spectral domain OCT instead of time domain OCT techniques [29]–[31]. Due to high axial scan velocities, high resolution and better reproducibility, spectral domain OCT has been proposed to be useful even for detecting subtle RNFL changes occurring independent of clinically manifest optic neuritis. [32] However, in contrast, a recent study in MS patients without optic neuritis did not detect RNFL thinning over a time-course of two years although high resolution spectral domain OCT was applied. [33].

When comparing baseline parameters of the patients included into our study, it was seen that treatment with IFN-β was more frequently started in patients with high numbers of clinically silent MRI lesions in the brain. This is explained by the observational design of the study and could be circumvented by blinding the treatment but this would not be in accordance with ethical considerations of not withholding approved treatments from patients. Additionally, longer VEP latencies were observed in the group receiving later IFN-β treatment which might also have influenced the decision to start with DMT on the one hand, and on the other, might have influenced the results of the final outcome parameter comparison. In a recent study, prolonged VEP latency at disease onset as well as three months later has been shown to be associated with greater RNFL loss [34] indicating that demyelination during acute optic neuritis increases vulnerability and later degeneration of optic nerve axons. Results of a covariate test performed in a recent neuroprotection trial with erythropoietin [15] as well as in this present study, however, did not indicate a strong effect of baseline VEP latency prolongation on the amount of RNFL degeneration at week 16. All other baseline parameters assessed in our present study such as initial RNFL oedema or visual acuity did not show any significant differences between the groups indicating that severity of optic neuritis was not generally different.

With respect to visual impairment, duration of symptoms, and presence of predictive factors for development of MS, our cohort was recruited in a highly selected manner explaining why the severity of disease (as indicated by functional and electrophysiological parameters) was higher than that of another cohort of comparable size [34]. However, applying strict selection criteria for study participation restricted the numbers of patients available for inclusion, a limitation of this preliminary study which would require confirmation in a larger group of patients. Additionally, LOCF for replacing missing RNFL values of the final visit has been applied in 30% of our cohort. This is a procedure which although not ideal we consider to be acceptable for this special type of study since approximately 95% of the total RNFL loss takes place within the first three months following the onset of symptoms. [14] In contrast to the large cohort included into the optic neuritis corticosteroid treatment trial [35] we did not allow MS to be present at the time of inclusion but we requested CSF or MRI risk factors for future MS development [16]–[18] in order to select patients potentially responsive to DMT. However, the absence of any treatment effects on structural or functional parameters of vision induced by early IFN-β treatment in our study may be explained by one or more of the following: Firstly, the disease course of optic neuritis and the kinetics of neurodegeneration in particular might be too rapid [14] to allow DMT to exert any influence. Beneficial effects of DMT in previous trials in CIS patients have been shown with respect to future conversion into MS and later progression of disability [36] which represents effects related to disease dissemination rather than those on initial lesion recovery or neurodegeneration caused by the initial lesion. Secondly, neurodegeneration in the retina might in parts occur independently of full activation of the immune system and, therefore, might only be minimally affected by immunomodulatory treatment approaches. In support of this notion, recent findings in an animal model of optic neuritis suggest that retinal neurodegeneration precedes inflammatory infiltration of the optic nerves. In this study, prior to the development of a humoral immune response, early neurodegeneration following disease induction was detected occurring in parallel with alterations in the blood-brain barrier and local microglial activation within the retina. [37] Additionally, data from OCT and histological studies in MS patients point towards primary pathophysiological changes in the retina that are not fully explained by inflammation and demyelination of the optic nerve. [38]–[40].

In conclusion, the absence of any effects on neurodegeneration and recovery of function after isolated optic neuritis does not imply that IFN-β treatment is generally not efficient in terms of further dissemination of the disease and conversion into MS. However, the results of this study suggest that present anti-inflammatory/immunomodulatory therapies are probably insufficient to successfully protect against neuronal loss, and therefore primary neuroprotective agents might be necessary as an add-on therapy to counteract neurodegeneration occurring during the acute phase of the disease.
